# Quality of routine health and nutrition data in Ethiopia: A systematic review

**DOI:** 10.1371/journal.pone.0316498

**Published:** 2025-03-03

**Authors:** Taddese Alemu Zerfu, Tirsit Genye, Amare Abera Tareke

**Affiliations:** 1 International Food Policy Research Institute (IFPRI), Addis Ababa, Ethiopia; 2 Department of Biomedical Sciences, College of Medicine and Health Sciences, Wollo University, Dessie, Ethiopia; Bahir Dar University College of Medical and Health Sciences, ETHIOPIA

## Abstract

**Background:**

High-quality data are vital for informed decision-making, enhancing population health, and achieving comprehensive insights. However, there is limited understanding of the consistency and reliability of routine Health Management Information System (HMIS) including nutrition data across diverse regions in Ethiopia. This study systematically reviewed the existing literature to address these knowledge gaps.

**Methods:**

We systematically searched PubMed, HINARI, and Google Scholar for studies published from 2015 onwards to assess HMIS, including nutrition data quality in Ethiopia. The evaluations focused on completeness, consistency, and timeliness metrics defined by the WHO. We included diverse regional studies without indicator restrictions, prioritized data quality metrics as primary outcomes, and explored qualitative reasons for poor data quality as secondary outcomes.

**Results:**

Of the 1790 papers screened, 25 met the inclusion criteria. The completeness of reporting varied widely among studies (50%–100%), with only 21% (4 out of 19) exceeding 90%. The consistency ranged from 38.9% to 90.5%, with only 6% of studies reporting internal consistency above 90%. Other consistency issues included lack of external consistency, indicator discrepancies, and outliers. Timeliness ranged from 41.9% to 93.7%, with 54% of studies reporting below 80%. In addition to the lack of studies addressing nutrition data, the quality was no better than other components of HMIS. The major factors contributing to poor data quality were human resource shortages, insufficient capacity building, behavioural influences, and infrastructural deficits.

**Conclusion:**

The HMIS including nutrition data in Ethiopia, exhibited deficiencies in completeness, consistency, and timeliness, which were largely, attributed to capacity and resource constraints. Interventions should prioritize resource allocation, staff training, supervision, and feedback mechanisms to enhance data quality, thereby improving decision-making processes and population health outcomes.

## Introduction

Globally, public health endeavours aimed at enhancing community well-being through health status evaluation, policy formulation, and service delivery assurance [1,2]. Given that, over 40% of countries lack evidence of adherence to data quality assurance processes in health facility data [3], the potential consequences of relying on unreliable data are severe [4].

The Health Management Information system (HMIS) collects, stores, analyses, and evaluates health-related data and is crucial for planning, monitoring, and evaluation of health programs and interventions [5]. The quality of data generated from HMIS is essential for effective decision-making in the health system [6].

Noting the crucial role of high-quality data, the World Health Organization (WHO) developed metrics for data quality [7]. The dimensions are completeness, consistency, and timeliness, there has been growing recognition of the importance of data quality in HMIS [8]. Completeness is the extent to which all necessary data elements are recorded and reported. Data completeness applies to various levels of reporting, from health facilities to districts and districts to regional levels. Consistency assesses the coherence and reliability of the data, both internally across different data sources (accuracy) and externally with other sources [9]. On the other hand, the timeliness of data measures whether the reports were submitted before the specified deadline [10]. It measures the promptness with which data are collected, entered the system, and made available for decision-making purposes.

Despite the pivotal role of the HMIS in Ethiopia’s healthcare system, challenges persist in ensuring data quality across different regions and health facilities. These challenges include human resource shortages, inadequate training in data management practices, infrastructural limitations, and behavioural factors that influence data reporting practices among healthcare personnel. Addressing these challenges is essential for enhancing the reliability, accuracy, and utility of HMIS data, thereby strengthening the evidence base for healthcare policies and interventions [11,12].

This systematic review aims to analyse data quality in the HMIS across Ethiopia, synthesizing findings from 2015 to identify trends, challenges, and best practices in terms of completeness, consistency, and timeliness. It provides strategies for enhancing HMIS data quality, improving healthcare delivery, and achieving better health outcomes.

## Methods

### Literature search

PubMed, HINARI, and Google Scholar databases were systematically searched using keywords such as “HMIS”, “Health management information system”, “Data quality,” “Completeness,” “Accuracy,” “Timeliness,” “Ethiopia”, and related terms, combined with Boolean operators. The final search was performed on March 29, 2024. This review adheres to the Preferred Reporting Items for Systematic Reviews and Meta-Analysis (PRISMA) guidelines for comprehensive reporting of search and selection criteria [13].

### Inclusion and study selection

This review considered research studies conducted in Ethiopia from 2015 onwards that examined at least one aspect of HMIS data quality. Eligible works included both published and unpublished material at the national or regional level, written in English. Studies had to clearly address HMIS data quality dimensions without restrictions on specific indicators (tracers). Excluded were opinion pieces, pre-post intervention studies, evaluations solely of medical records, validation studies, and reports not focused on HMIS data quality. A rigorous two-stage screening process involved initial assessment of titles and abstracts, followed by full-text evaluation by two reviewers. Any discrepancies were resolved through consensus between the reviewers at each screening stage to ensure comprehensive inclusion based on predefined criteria.

### Data extraction

A standardized data extraction tool was developed following guidelines from the Cochrane Collaboration and the Center for Review and Dissemination. This tool captured study specifics such as authorship, publication year, study design, geographical region, and evaluated indicators, along with qualitative and quantitative main findings. Additionally, the tool facilitated quality assessment, particularly for observational studies using the Newcastle Ottawa Scale (NOS) [14]. Cross-sectional studies were categorized based on their methodological quality as Very Good (9–10 points), Good (7–8 points), Satisfactory (5–6 points), or Unsatisfactory (0–4 points). This rigorous approach ensured systematic and comprehensive extraction of relevant data to support the review’s objectives and maintain robustness in synthesizing study outcomes. Two reviewers performed the data extraction process.

### Outcome measurement and data synthesis

Our primary objective was to evaluate data quality, assessed through metrics including completeness, consistency, and timeliness, defined by WHO standards [7]. Secondary outcomes involved qualitative findings, aiding in understanding factors contributing to poor data quality. Data extraction involved summarizing outcomes into tables, facilitating a systematic presentation of findings. The systematic review results were synthesized both narratively and through tabular formats, ensuring comprehensive coverage and clarity in reporting the study’s outcomes and implications. We failed to pool the results due to heterogeneity of variables and inconsistent reporting.

## Results

### Search results

**Fig 1:**
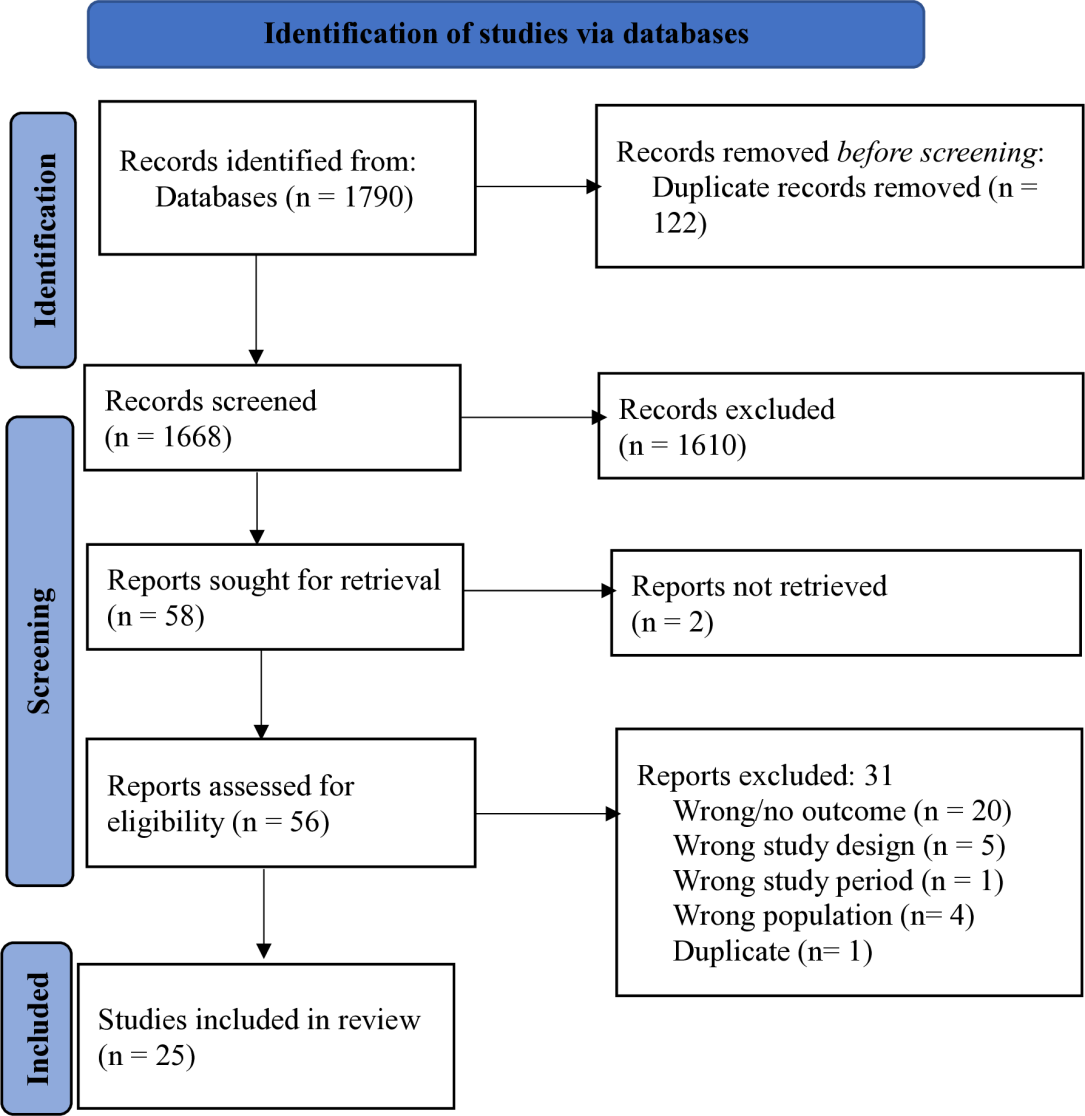
The PRISMA flow chart for study selection and screening.

The initial search across databases identified 1790 papers, from which 122 duplicates were removed, leaving 1668 articles for screening. Following title and abstract evaluation, 1612 articles were excluded due to irrelevance, resulting in 56 articles for full-text assessment. Ultimately, 25 articles [15–39] met the inclusion criteria for the review. Exclusion reasons included lack of outcomes related to data quality dimensions (n = 20), focus on evaluating HMIS tool effectiveness (n = 5), inappropriate population (mostly related to medical records) (n = 4), study period mismatch (n = 1), and duplicate studies (conducted on the same population), Fig 1.

### Characteristics of the included studies

Eight studies (32%) were conducted either nationally or across multiple regions [19,26,28,29,33,34,36,38]. Specifically, four studies focused in the Amhara region [21,22,30,31], three studies in South Nations Nationalities and People’s Region (SNNPR) and Oromia region [16,27,39], two studies each for Addis Ababa [15,23] and Tigray [17,25]. Additionally, there were single studies from each region (Somali [35], Harari [18], and Benishangul Gumuz [32]). The studies were published between 2016 and 2024, reflecting recent assessments of HMIS data quality across Ethiopia’s diverse geographical and administrative contexts.

Most of the studies were cross-sectional studies, 11(44%) studies [15,21,25–28,30–32,34,37] used mixed design to incorporate qualitative findings as well. Almost all studies assessed maternal and child health indicators for assessment of data quality, two studies [18,23] failed to specify indicators used to determine data quality. Most included studies reported the following indicators: first antenatal visit (ANC1), fourth antenatal visit (ANC4), skilled birth attendance (SBA), postnatal care (PNC), child immunizations, and contraceptives. Few studies used the WHO tracers (indicators of data quality), and no study used all five indicators.

Most of the included studies compared the HMIS report and the registries (source document). Registers are records that are filled by health professionals at different units. Usually, these documents are filled out daily. Other studies compared the HMIS report with WHO/UN estimates of immunization [26], HMIS report with population-based surveys [16,19,33,36], the HMIS report with Emergency Obstetrics and Newborn Care Assessment data [38], and the HMIS report with JSI coverage data as well as linked to biomarkers using serosurvey for immunizations [29]. The characteristics of the included studies are summarized in Table 1.

**Table 1:** Characteristics of included studies.

	Author	Year	Study area	Qualitative	Design	Indicators	Completeness	Consistency	timeliness	Quality
**1**	Shama et al.	2021	Harari	No	Cross-sectional	Not specified	Yes	Yes	Yes	8
**2**	Endriyas et al.	2019	SNNPR	Yes	Cross-sectional	ANC4, SBA, PNC, total malaria, confirmed malaria, TB, fully immunized, institutional maternal death, and SAM		Yes		8
**3**	Pond et al.	2021	National	Yes	Cross sectional	DPT3		Yes		9
**4**	Ouedraogo et al.	2019	Oromia	No	Cross-sectional	ANC1, ANC4, SBA, PNC, DPT1, DPT3, Malaria, stillbirth	Yes	Yes	Yes	9
**5**	Worku et al.	2022	National	No	Cross-sectional	BCG, OPV0, DPT, PCV, Rota, measles, Vitamin A, fully immunized	Yes	Yes		8
**6**	Kebede et al.	2020	Oromia	Yes	Cross-sectional	Penta1, ANC, measles, FP, SBA, contraceptives, OPD visits, PMTCT, PNC	Yes	Yes	Yes	9
**7**	Worku et al.	2022	National	No	Cross sectional	SBA	Yes	Yes		7
**8**	Derbew et al.	2024	Tigray	No	Cross-sectional	Live birth, ANC, PNC, Penta, neonatal deaths, still birth, under-five death	Yes	Yes		7
**9**	Travassos et al.	2016	Tigray, Afar, SNNPR	No	Cross sectional	Penta3, tetanus toxoid		Yes		8
**10**	Adane et al.	2021	National	No	Cross sectional	ANC1, ANC4, SBA, PNC, early neonatal death at community, early institutional neonatal death, births, Penta3, Measles, fully immunized, malaria, TB	Yes	Yes		8
**11**	Solomon et al.	2021	SNNPR	No	Cross-sectional	ANC4, SBA, Penta3, PMTCT, TB, malaria, Contraceptive accepters’ rate	Yes	Yes	Yes	9
**12**	Arsenault et al.	2021	National	No	Cross sectional	Deliveries and C-section		Yes		6
**13**	Chekol et al.	2023	Amhara	Yes	Cross-sectional	ANC4, CAR, institutional delivery, Penta 3, PMTCT, TB cure rate, and confirmed cases of malaria	Yes	Yes	Yes	9
**14**	Haftu et al.	2021	Addis Ababa	Yes	Mixed	DM, HTN, ANC1, ANC4, Malaria, Penata3, VCT, Prescription, TT1, Contraceptive, TB,	Yes	Yes	Yes	8
**15**	Getachew et al.	2022	SNNPR	No	Cross-sectional	ANC tested for syphilis, PNC, SBA, fully immunized, PMTCT, TB, malaria, contraceptive	Yes	Yes	Yes	9
**16**	Ayele et al.	2021	Addis Ababa	No	Cross sectional	Not specified	Yes	Yes	Yes	5
**17**	Gobena et al.	2022	Somali	No	Cross-sectional	ANC, delivery, immunization, VCT, impatient, TB, adult, pneumonia, SAM	Yes	Yes		9
**18**	Tilahun et al.	2021	Amhara	Yes	Cross-sectional	ANC1, FP, delivery, malaria, HIV^ + ^, pneumonia	Yes	Yes	Yes	5
**19**	Tilahun et al.	2022	Benishangul Gumuz	Yes	Mixed	ANC1, FP, delivery, malaria, HIV^ + ^, pneumonia	Yes	Yes		7
**20**	Madebo et al.	2021	Amhara	Yes	Mixed	BCG, Penta1, penta3, fully immunized, measles	Yes	Yes		9
**21**	Wordofa et al.	2022	Oromia and Gambella	Yes	Cross-sectional	contraceptive, ANC4, SBA, PNC, penta3, measles, malaria, pneumonia	Yes	Yes	Yes	7
**22**	Gebreslassie et al.	2020	Tigray	Yes	Cross-sectional	SBA, Penta three vaccination, family planning use and pneumonia in under five children	Yes	Yes		3
**23**	Adane et al.	2021	Afar, Oromia, SNNPR, Tigray	Yes	Cross-sectional	ANC, early neonatal death at community, early institutional neonatal death, births, Penta3, Measles, fully immunized, malaria, TB	Yes	Yes	Yes	8
**24**	Demeke	2022	Amhara	No	Cross-sectional	ANC4, SBA, Penta, TB, PMTCT, Malaria, Contraceptives	Yes	Yes	Yes	9
**25**	Nesru et al.	2017	Oromia	No	Cross-sectional	Family planning, ANC4, SBA, Penta3, Growth monitoring, impatient discharge, malaria positive	Yes	Yes	Yes	9

*ANC: Antenatal care, SBA: Skilled birth attendant, TB: Tuberculosis, PMTCT: Prevention of mother-to-child transmission of HIV, PNC: Postnatal care, BCG: Bacillus Calamite Guerin, FP: Family planning, SAM: Severe acute malnutrition, VCT: Voluntary counseling and testing, DM: Diabetes Mellitus, HTN: Hypertension, CAR: Contraceptive acceptance rate, TT: Tetanus toxoid, OPD: Outpatient department, OPV: oral polio vaccine, DPT Diphtheria-Tetanus-Pertussis, SNNPR: South Nations Nationalities and People’s Region Mixed - includes qualitative component*

#### Completeness of routine health data.

In nineteen studies (76%), completeness was the primary focus [15,16,18–25,27,28,31–36,39]. According to WHO guidelines [40], encompasses two dimensions: the completeness of reports reaching the next level and the completeness of indicator data. WHO-defined indicators include ANC first visit, 3rd dose of Diphtheria-Tetanus-Pertussis (DTP) containing vaccine, newly initiated antiretroviral therapy (ART), notified cases of all forms of tuberculosis (TB), and confirmed malaria cases [40]. Among the studies addressing completeness, only six (32%) specifically examined reporting completeness [16,22,28,32,34,39], with the remaining studies concentrating on the completeness of indicator data, Table 2.

**Table 2:** Summary of main findings.

N_o_	Author	Main findings
**1**	Shama et al. 2021^a^	Overall data quality = 51.4%, timeliness = 93.7%, completeness = 60%, accuracy = 58.1%. factors associated with data quality were the type of facility, presence of trained person, and feedback
**2**	Endriyas et al. 2019^a^	Maternal death and skilled delivery were reported accurately, but ANC4, early PNC, and fully immunized children were often over-reported. Conversely, total malaria, TB case detection, and severe acute malnutrition were consistently under-reported due to mobile phone reporting without documentation, poor registration, and inadequate supervision, among other factors.
**3**	Pond et al. 2021^b^	In 2016 admin DPT3 was 96%, EDHS = 53%, and Documents = 57%. In 2019 admin DPT3 coverage was 96%, miniDHS 2019 61%. Reasons: Inadequate staff training, uncertain denominators, and “false [over]reporting” for recognition and promotion
**4**	Ouedraogo et al. 2019^c^	Timeliness = 51.3%, completeness = 52.7%, accuracy = 4.0% of outliers. HMIS reports higher values than survey results for most indicators. There was good agreement between stillbirth rates and malaria during pregnancy
**5**	Worku et al. 2022^c^	Completeness = 85%, Accurately reporting = 63% Reasons: Staffing issues (shortage, absence), poor understanding of the data element by health workers and negligence were top three reasons for incomplete source document
**6**	Kebede et al 2020^a^	Completeness = 86%, timeliness = 72.2%, accuracy = 48%, Low data quality in all dimensions, which is below the national standards. Reasons; poor support of management, lack of accountability for the false report, poor supportive supervision, and lack of separate and responsible unit for health information management
**7**	Worku et al. 2022^c^	Completeness = 73%, Accuracy = 74%, the data quality of all maternal service indicators is below the national standard.
**8**	Derbew et al. 2024^d^	Live births, child health service indicators, and child health events were more erratically reported in the three data sources. There was discordance
**9**	Travassos et al. 2016^e^	Penta3 admin report = 85.2%, JSI coverage data = 52.2%, Sero-survey = 63.9%. Administrative data overestimated the service coverage
**10**	Adane et al. 2020^c^	For many indicators, denominators were based on poor-quality population data estimates. Data on vaccinations had relatively good internal consistency. But, fully vaccinated HMIS 89%, EDHS 39%. Indicators on child nutrition, malaria, and tuberculosis were less consistent. One in eight early neonatal deaths was reported in HMIS
**11**	Solomon et al. 2021^a^	Accuracy = 76%, Completeness = 83.3%, Timeliness 88.4%, Over all data quality = 82.5%. ANC4 (19%), Contraceptives (16%), penta3 (15%), PMTCT (14%) over-reported.
**12**	Arsenault et al. 2021f	65.7% of facilities have good HMIS reporting of deliveries, deliveries are more likely to be over-reported and C-sections under-reported in most regions. Good reporting is common in urban areas, public facilities, and hospitals compared with health centers.
**13**	Chekol et al. 2023^a^	Accuracy = 74%, completeness = 70%, timeliness = 78%, Overall data quality = 74%. Factors; complexity of HMIS format, problem-solving skills, unable to take responsibility, lack of personnel and training, poor feedback mechanism, delay in completing data records
**14**	Haftu et al. 2021^a^	Overall data quality = 76.2%,Accuracy = 77%, Timeliness = 41.9%, Completeness 91.2%. Factors: Health professionals motivation is associated with data quality. Lack of adequate Health information system task competence, non-functional PMT, and lack of supervision were also commonly reported reasons for poor data quality.
**15**	Getachew et al. 2022^a^	Data quality = 83%, Accuracy = 79%, completeness = 86%, timeliness = 84%. Supportive supervision, checking accuracy, filling registrations and confidence level positively associated with data quality.
**16**	Ayele et al 2021^a^	Overall date quality = 57.9%, Accuracy = 69.6%, Completeness = 49.5%, Timeliness = 56%. Factors: Supportive supervision and mentorship
**17**	Gobena et al. 2022^a^	Data accuracy = 88.1%, completeness = 75.8%, data recording value given by their immediate supervisors was a strong predictor.
**18**	Tilahun B et al. 2021^a^	Accuracy = 90.5%, Completeness = 96.2%, Timeliness = 66.7%, Factors: valuing data, getting staff training, being a patriotic staff, coaching, supportive supervision, and peer-to-peer learning, incentive, establishing accountability, and staff turnover
**19**	Tilahun B et al. 2022^a^	Completeness = 75%, ANC1 (2%), family planning (1%), malaria (6%), and pneumonia (27%) over reported. Delivery (by 4%) and HIV^ +^ (by 42%) under reported. Qualitative findings: Individual motivation, capacity building, commitment, digital literacy, infrastructure, healthcare information system resources, supportive supervision, healthcare data, assigning the right person, and system regulation.
**20**	Madebo et al. 2021^a^	Over-reporting of 44%, 46%, 40%, 37%, and 38% for BCG, Penta1, Penta3, Measles, and full immunization. Factors: Supervision, availability of recording and reporting tools, training, motivation, attitudes towards healthcare data, hard-to-reach areas, and manual documentation
**21**	Wordofa et al. 2022^a^	Delivery service (98%), Penta-3 (96%), and Measles (94%) reported in acceptable level. The others were out of the acceptable range, indicating the presence of over-reporting. Report completeness was 100%. Timeliness, 53.5% of the report in Digalu-Tijo and 79% in Godere
**22**	Gebreslassie et al. 2020^a^	Register and report completeness was 53.5% and 56.3% respectively. Reasons for incompleteness: a shortage of staff, poor of understanding the data element, presence of other vertical reporting requirements, and the recording tool is not designed as user-friendly. Consistency = 38.9%. Reasons: data entry errors, arithmetic errors, and lack of emphasis on data accuracy.
**23**	Adane et al. 2022^a^	The majority of indicators had gaps in reporting. Maternal health and postnatal indicators had the most gaps in reporting. Completeness of reporting for nutrition was also low, at slightly over 50% for the facility-months reviewed. Completeness was much higher for immunization. Timeliness was over 90% for maternal health indicators. Maternal and immunization indicators had lower proportions of reports within the range for acceptable quality. Varying levels of over-reporting were observed in all service coverage indicators, but not for severe acute malnutrition. Factors: the number and complexity of forms, parallel reporting, language issues (especially in health posts), inappropriate denominators, access to computers and internet, shortage of supplies, electricity, human resource shortages, lack of training and staff turnover, fear of reporting low service coverage or unwanted results, Supervision, rarely focus on data quality, difficulties understanding the registration and other forms, performance management, and basics of data entry and analysis, lack of knowledge and skill on checking data quality, and lack of interest in RHIS resulting from low personal motivation and work overload.
**24**	Kassa 2021^a^	Completeness = 86.4%, Timeliness = 85.7%, Accuracy = 76.2%, Overall data quality = 82.8%. ANC4, SBA, penta3, PMTCT, and total contraceptive were over-reported. TB cure rate and confirmed malaria cases were under reported. Conducting accuracy tests, the presence of standard indicators with their definition, Data management support, feedback, and training were significantly associated.
**25**	Nesru et al.^a^	Overall data quality = 81%, Accuracy = 57.2% which is lower than the national target for data accuracy. From indicators low accuracy was observed in Family planning (35.6%), Antenatal care four visit (40.4%). Over-reporting was higher (32.2%) than under-reporting (10.6%) in all facilities. Completeness and report timeliness were 94.9% and 85% respectively and this attains the national target for both.

*The lower-case alphabet a-f indicates the comparison of HMIS.* ^***a***^***registers(source documents),*** ^***b***^***UN reports for national immunization coverage,*** ^***c***^***population based surveys,*** ^***d***^***Family folder and HMIS and household survey,*** ^***e***^***JSI coverage data and sero-survey for pentavalent 3,*** ^***f***^***2016 Emergency Obstetrics and Newborn Care Assessment,***

***ANC: Antenatal care, SBA: Skilled birth attendant, TB: Tuberculosis, PMTCT: Prevention of mother-to-child transmission of HIV, PNC: Postnatal care, DPT:*** *Diphtheria-Tetanus-Pertussis, HMIS: Health management information system, EDHS: Ethiopian Demographic and Health Survey.*

The degree of completeness in reporting varied significantly among studies, with reported rates ranging from less than 50% to 100%. For example, district health offices in Jimma zone received 52.8% complete reports [16], while rates were 75% in the Benshangul Gumuz region [32], 86.4% in east Gojjam Zone [22], and 100% in two districts across Oromia and Gambella regions [34]. Adane et al [28] observed varying completeness levels across health services; for instance, immunization had higher completeness rates compared to other services, where rates fell below 50%.

Fourteen studies assessed data completeness using various service indicators, but the summary measures were inconsistent. Most studies lacked denominators when reporting completeness percentages, which hindered result aggregation. Only three studies, conducted in Addis Ababa [15], the Oromia region [39], and the Amhara region [31], reported completeness exceeding 90%. Four studies reported completeness rates between 80 and 90% [19,20,24,27], while the remaining studies fell below 80%. With a completeness benchmark of 90%, only three out of the 14 studies (21%) indicated satisfactory data completeness in Ethiopia.

When considering both report and document completeness collectively, only four out of 19 studies (21%) reported completeness rates exceeding 90%. Notably, completeness rates reached as low as 53.6% in the Tigray region [25]. The diversity in reporting practices, utilization of different indicators, and regional disparities highlight significant gaps in data quality, particularly concerning completeness, in Ethiopia.

#### Consistency of routine health data.

All studies included in the analysis provided information on data consistency, either qualitatively or quantitatively. Internal consistency was assessed in eighteen studies, which compared source documents (registers) with reported data using various indicators, detailed in the respective studies. Additionally, seven studies examined external consistency, evaluating the alignment of reported data with other independent sources or benchmarks. These assessments aimed to understand discrepancies, such as over-reporting or under-reporting, enhancing the overall reliability of data accuracy evaluations.

*Internal consistency (accuracy):* Internal consistency was examined in eighteen studies [15,17–25,27,28,30–35,37,39] by comparing source documents (registers) with reports using various indicators (as detailed in the respective studies). However, inconsistent reporting of denominators hindered the quantitative pooling of accuracy levels. Accuracy percentages reported ranged widely from 38.9% [25] to 90.5% [31], with one study exceeding 90% accuracy [31], and another falling between 80% and 90% [35]. Some studies noted discrepancies such as over-reporting in certain indicators and under-reporting in others, adding complexity to accuracy assessments.

The prevailing trend indicates significant over-reporting of immunizations and maternal and child health services across studies [16,19,22,24,27,28,30,34,37,39]. Child immunizations often exceeded documented figures in registers, consistently observed in all included studies. Similarly, maternal health indicators like ANC, PNC, family planning, and deliveries were frequently reported in excess [15,22,24,27,28,32,34,37,39]. Notably, one study highlighted deliveries in HMIS being reported 30.89 times more than recorded in registries [38]. While some studies reported acceptable levels of accuracy [34,37], instances of under-reporting were also noted [32]. Conversely, adverse events and disease states such as tuberculosis, pneumonia, severe acute malnutrition, and malaria were consistently under-reported [22,28,32,37,39], highlighting discrepancies in reporting accuracy across health indicators.

*External consistency:* Seven studies assessed the external consistency of HMIS reports by comparing them with various sources, including WHO/UN estimates of immunization [26], population-based surveys [16,19,33,36], Emergency Obstetrics and Newborn Care Assessment data [38], and JSI coverage data, as well as biomarker-linked serosurveys for immunizations [29]. The findings consistently revealed that the HMIS overestimated indicators for maternal and child health. For instance, comparisons with the 2016 and 2019 Ethiopian Demographic and Health Surveys (EDHS) revealed that HMIS reported significantly higher DPT3 coverage: 96% versus 53% in 2016 and 96% versus 61% in the 2019 mini-DHS [29]. Similar discrepancies were noted for the other vaccines and fully vaccinated children. Despite inflated vaccination reports, one in eight early neonatal deaths have been reported [36]. Although the HMIS aligned somewhat with population-based surveys, it consistently reported higher values. This trend persisted across studies, where maternal health services were more likely to be over-reported (20%) than under-reported (6%) [33]. While the HMIS tended to exaggerate maternal and child health indicators, certain outcomes, such as early neonatal deaths, were under-reported.

**Consistency with related indicators:** Consistency with related indicators: Several studies explored the coherence between related indicators, revealing significant disparities. For example, in certain districts, there was a 30%–63% higher attendance for first ANC visits compared to children receiving their first dose of DPT1 [16]]. Additionally, 44% of facilities had ratios greater than 1 for ANC4/ANC1 and DPT3/DPT1, indicating more cases of ANC4 and DPT3 than their respective denominators [16]. The inconsistency persisted across comparisons of syphilis testing and ANC1 [34], total births, PNC, and DPT [36], underscoring inconsistencies in related indicator comparisons.

**Consistency over time:** Three studies [16,34,36] investigated trends in maternal and child health service utilization over time, revealing an overall increase. These indicators remained consistent at the national level [36] or within specific districts [16,34]. However, variability was noted in neonatal and child health indicators across less than half of Ethiopia’s regions, suggesting inconsistent trends over time.

**Outliers:** Two studies utilized outliers to evaluate data quality. One study detected 4.03% outliers [16] while the other study identified a mix of extreme and moderate outliers across various indicators [36].

#### Timeliness of routine health and nutrition data.

Thirteen studies [15,16,18,20–24,27,28,31,34,39] assessed the timeliness of reports, revealing inconsistent results ranging from 41.87% [15] to 93.7% [18]. Two studies reported timeliness above 90% [18,28], while four studies fell between 80% and 90% [20,22,24,39]. However, 54% of the studies reported timeliness below 80%, with five out of these seven studies indicating timeliness below 70%.

#### Overall quality of routine health data.

Seven studies [15,18,21–24,39] synthesized various indicators to assess overall data quality. None of the studies reported data quality equal to or greater than 90%. Three studies reported data quality above 80% [22,24,39], while two studies reported 74% [21] and 76% [15]. The remaining two studies reported lower data quality percentages of 58% [23] and 51% [18]. These findings collectively indicate unsatisfactory overall data quality, falling short of the expected standard of 90%. The summary of findings from the included studies is shown in Table 2.

#### Nutrition data quality.

Five studies [19,28,35–37] evaluated data quality for nutrition indicators, revealing lower quality compared to other child and maternal health indicators. Severe acute malnutrition was notably under-reported [28,36,37], with accuracy levels ranging from 55% [37]to 97% [35]. Nutrition indicators also showed lower completeness rates, with only half of the reports submitted on time [28,36,37]. Significant gaps in external consistency were observed, particularly for deworming and vitamin A supplementation [36]. Overall, data quality for nutrition indicators in HMIS did not meet the standards seen in other indicators. Since nutrition indicators are not part of WHO’s quality assessment, limited research has evaluated their quality, suggesting potential for further data quality issues in this area. The main findings regarding nutrition data quality are shown in Table 3

**Table 3:** Main findings of studies on nutrition data quality.

	Author	Nutrition outcome	Main findings
**1**	Endriyas et al. 2019	SAM	The accuracy level of SAM was (54.6%) within 10% precision. The verification factor indicated there was 2% under-reporting of SAM.
**2**	Worku et al. 2022	Vitamin A	The findings are 85% of the health institutions had complete source document, and 63% of the health institutions were accurately reporting.
**3**	Adane et al. 2020	Vitamin A, Deworming, Severe acute malnutrition, and Growth monitoring	Nutrition data were generally complete. Child nutrition indicators exhibited inconsistent trends in less than half of the areas. Vitamin A supplementation and deworming showed both extreme and moderate outliers. External consistency for nutrition indicators, such as deworming, was notably poor, with disparities between national surveys and HMIS reports.
**4**	Gobena et al. 2022	SAM	The data accuracy and content completeness of SAM were 96.7% and 70.4% respectively
**5**	Adane et al. 2021	SAM, deworming, Vitamin A growth monitoring	Nutrition reporting completeness was slightly over 50% for reviewed facility months. Just over half of nutrition indicator reports were timely, and accuracy levels varied within acceptable ranges.
**6**	Nesru et al. 2017	Growth monitoring	Accuracy = 39.8%, over-reporting = 51%, under-reporting = 9.2%,

*SAM: Severe acute malnutrition, HMIS: Health management information system*

#### Challenges undermining routine health and nutrition data quality.

Supportive supervision and feedback were frequently cited factors influencing data quality in eleven studies [15,18,20,21,23,27,28,30–32,37]. Lack of training was identified in seven studies [18,21,22,26,28,30–32], while the absence of data management oversight was noted in six studies [19,21,25,27,28,31].

Various challenges influencing data quality were highlighted across the studies, including issues such as mobile phone reporting without proper documentation, inadequate registration practices, illegible data, and negligence. Competition manipulation, insufficient competency, failure to review performance, and limited experience sharing were also significant concerns. Neglecting institutional data quality assessments, lack of commitment, and the absence of essential tools, such as tally sheets, further compounded these challenges. Moreover, issues such as poor integration of data systems, the complexity of forms, and parallel reporting added to the complexity alongside language barriers in health posts and inappropriate denominators.

Additionally, factors such as limited access to computers, the Internet, electricity, fear of reporting low service coverage, and difficulties in understanding registration forms were identified. Inadequate knowledge and skills in data quality checks, lack of interest in Routine Health Information Systems due to low motivation and work overload, and false (over)reporting for recognition and promotion were also noted. Moreover, the absence of accountability for false reporting, peer-to-peer learning, incentives, commitment to digital literacy, challenges specific to urban areas, and issues in higher-tier systems contribute to the overall complexity of ensuring robust data quality in HMIS [15,18,20–23,26–28,30–33,35,37,38].

## Discussion

We systematically reviewed databases to evaluate routine health and nutrition data quality across Ethiopia, focusing on completeness, consistency, and timeliness within the HMIS. Our analysis revealed pervasive shortcomings: HMIS data consistently fell short of the established standards, particularly in terms of completeness and consistency. Although timeliness showed some improvement, there were clear opportunities for improvement. Nutritional data present significant challenges, highlighting the critical need for improved accuracy. Overall, our findings emphasize the urgent need for systematic enhancements in data governance and reporting practices to bolster the reliability of healthcare information in Ethiopia.

High-quality healthcare data are indispensable for informed decision-making at all levels of the health system. It supports strategic policy formulation and efficient resource allocation and enhances decision-making by consolidating lower-level data. Conversely, poor data quality undermines the decision-making processes, leading to suboptimal outcomes [41]. Enhanced data quality empowers organizations to minimize uncertainty, boost productivity, and deliver healthcare services more effectively [3]. Furthermore, it mitigates risks to organizational reputation and lowers healthcare costs with potentially significant financial benefits [42].

The assessment of data quality within Ethiopia’s HMIS reveals multifaceted challenges and important insights into the reliability of health and nutrition indicators. Maternal and child health indicators were prominently featured across the studies, reflecting their critical role in assessing data quality. However, variability in indicator specification has been noted, with some studies omitting specific metrics, which complicates standardized assessments and global comparisons. The limited use of comprehensive WHO tracers further underscores the inconsistencies in applying standardized indicators across evaluations, highlighting the need for improved uniformity in reporting practices.

Completeness rates varied significantly across regions and health services in Ethiopia, ranging from under 50% in some districts to 100% in selected areas of Oromia and Gambella. This disparity points to an unevenness in reporting practices and challenges within specific health sectors. Notably, immunization services often demonstrated higher completeness rates than other health indicators, reflecting the varying priorities and capacities in data reporting. However, inconsistent reporting practices and the use of different metrics hinder their ability to uniformly assess and benchmark data quality against international standards.

When comparing Ethiopia’s data completeness challenges with other countries like Ghana and Tanzania, parallels emerge in disparities across regions and health sectors. Ghana faces under reporting and inconsistencies in certain districts, influenced by logistical challenges and varying capacities in data management [43]. Similarly, Tanzania contends with regional variations due to infrastructure limitations and inadequate healthcare personnel training [44]. In contrast, Kenya, Nigeria and Bangladesh have improved completeness through technology and training, emphasizing standardized reporting and enhancing data reliability for informed decision-making and improved health outcomes [45–47].

Internal consistency assessments, conducted in eighteen studies, compared reported data with source documents (registers), revealing a wide range of accuracy levels. These assessments highlighted instances of over-reporting in immunizations and maternal health services, where reported figures exceeded those recorded in the registries. This discrepancy suggests potential inaccuracies in data entry or reporting biases that could skew the overall health assessments and resource allocation decisions.

External consistency evaluations, explored in seven studies, scrutinized the alignment of HMIS data with independent sources, such as WHO/UN estimates and population-based surveys. The findings consistently indicated over estimations in maternal and child health indicators compared with benchmarks that are more reliable. Disparities were particularly evident in vaccination coverage, where HMIS reports often exceeded demographic and health survey findings. Such inconsistencies underscore the challenges of ensuring data reliability across different health sectors and highlight the importance of robust validation mechanisms to enhance data accuracy.

Ethiopia’s HMIS data, as assessed through external consistency evaluations, shows notable discrepancies compared to other countries. Similar evaluations in countries like Kenya and Nigeria have also revealed challenges with over estimations in maternal and child health indicators within their respective HMIS. However, while these countries have implemented strategies to improve data accuracy, such as enhanced validation processes and standardized reporting guidelines [47], Ethiopia faces ongoing challenges in aligning HMIS data with more reliable benchmarks like WHO/UN estimates and demographic surveys. These disparities underscore the broader global challenge of ensuring accurate health data reporting across diverse health systems and highlight the critical need for strengthened validation mechanisms and improved data governance practices to enhance data reliability and inform evidence-based decision-making in healthcare.

There is a need to enhance the completeness of data in Ethiopia’s routine HMIS. Although a few studies have achieved completeness rates of >  90%, most have not met this standard, highlighting the need for improvement. Standardizing reporting practices and ensuring the consistent use of summary measures could help address regional variations and enable more accurate assessments of the quality of health data nationwide. These efforts are essential for improving the reliability and usefulness of health information for evidence-based decision-making and policy development.

Compared with other countries, countries like Uganda have made strides in achieving high completeness rates by implementing standardized reporting practices and rigorous data validation mechanisms [48]. In contrast, Ethiopia faces greater disparities across its regions and health services, where achieving uniform completeness remains a challenge due to inconsistent reporting practices and varying capacities in data management. While all these countries prioritize enhancing data quality for evidence-based decision-making, Ethiopia can benefit from adopting similar strategies to improve the reliability and utility of its health information nationwide.

Outlier analysis in two studies provided additional insights into data quality issues, identifying extreme and moderate deviations in the reported indicators. This methodological approach helps identify anomalies that could distort the overall assessment of health data reliability and accuracy, thereby informing targeted interventions to improve data management practices.

The timeliness of data reporting, as assessed in 13 studies, varies widely across different health facilities and regions. While some reported timeliness rates above 90%, others fell below acceptable thresholds, indicating systemic challenges in ensuring timely data submission. Addressing these challenges is critical for maintaining up-to-date health information that is crucial for effective planning and response to public health needs.

Countries like Ghana and Nigeria also employ outlier analysis to pinpoint discrepancies that affect data reliability, supporting targeted interventions to enhance reporting accuracy [49]. However, Ethiopia’s timeliness of data reporting, as evidenced by 13 studies, shows significant variability compared to countries like Rwanda and Uganda [50,51], where efforts to streamline reporting processes have resulted in consistently higher timeliness rates across health facilities. Addressing these disparities through improved infrastructure and standardized reporting protocols is essential for Ethiopia to maintain timely and accurate health information, crucial for effective public health planning and response.

The results of seven studies assessing data quality in Ethiopia underscore persistent shortcomings, with none meeting the desired 90% standard and many falling below 80%. These deficiencies across accuracy, completeness, and timeliness highlight urgent needs for systematic improvements in the country’s health information systems to enhance reliability and support informed decision-making and resource allocation.

Compared with countries like Kenya and Rwanda, Ethiopia faces greater challenges in data quality [51,52]. These nations have implemented robust data governance and reporting strategies, resulting in higher overall data quality scores and consistent adherence to international standards. These comparisons emphasize the imperative for Ethiopia to adopt similar systematic improvements in its health information systems to ensure reliable health data for effective public health management.

This study has several strengths. It adopts a comprehensive approach by reviewing multiple studies and providing a broad and detailed analysis of data quality within Ethiopia’s HMIS. It assesses both internal and external consistency, outlier analyses, and timeliness of data reporting, thus offering diverse perspectives on data reliability. This multifaceted approach enhances the robustness and depth of the findings, allowing for a nuanced understanding of the complexities and challenges within the HMIS. Additionally, by synthesizing findings across various health indicators and regions, this study contributes to a more holistic view of the strengths and weaknesses of Ethiopia’s health information systems.

However, this study is not free of limitations, owing to the variability in methodologies among the reviewed literature, which may hinder the direct comparability of findings. Each study included in the review may have employed different criteria, metrics, or analytical techniques, potentially skewing the synthesis of the results. Moreover, reliance on published studies introduces publication bias, as unpublished data and negative results may not be represented, potentially influencing the overall assessment of data quality in Ethiopia’s HMIS. Furthermore, the geographical focus on specific regions and health indicators within Ethiopia limits the generalizability of our findings to the entire country. Variations in health service delivery and data management practices across different regions may not be fully captured, thus restricting a comprehensive understanding of nationwide data quality challenges. Given the recent instability of the country and internal displacement of populations, coverage data might be influenced. Some areas, which have incoming population, may have above 100% coverage, this scenario might affect recent studies and external consistency results might be influenced.

In conclusion, the synthesis of findings underscores significant challenges in data quality within Ethiopia’s HMIS, marked by inconsistencies in reporting practices, regional disparities, and limitations in standardization across health indicators. Despite these challenges, this study’s comprehensive review highlights opportunities for improvement, including enhanced training, standardized reporting protocols, and strengthened data management oversight. Addressing these issues is crucial for enhancing the reliability and utility of health data, and ultimately facilitating more effective policy formulation, resource allocation, and health interventions tailored to the diverse needs of Ethiopia’s population.

## Supporting information

S1 Checklist PRISMA_2020_checklist. (DOCX)

S2 File Risk of bias analysis. (DOCX)
