# Supplementary material for: Quality of routine health and nutrition data in Ethiopia: A systematic review
Source: PLoS One. 2025 Mar 3;20(3):e0316498. doi: 10.1371/journal.pone.0316498 (PMC11875334; doi:10.1371/journal.pone.0316498)
Supplement: S2 File — (DOCX) [file pone.0316498.s002.docx]

**Quality of Routine Health and Nutrition Data in Ethiopia: A Systematic Review**

Taddese Alemu Zerfu^1*^, Tirsit Genye^1^, and Amare Abera Tareke^1,2^

Risk of bias analysis – The Newcastle Ottawa Scale

|  | Representativeness of the sample | Sample size | Non-respondents | Ascertainment of the exposure (**) | Comparability of subjects | Assessment of outcome (**) | Statistical test | score |
| --- | --- | --- | --- | --- | --- | --- | --- | --- |
| Shama et al. 2021 | * | * |  | ** | * | * | * | 8 |
| Endriyas et al. 2019 | * | * | * | ** |  | * | * | 8 |
| Pond et al. 2021 | * | * | * | ** | * | * | * | 9 |
| Ouedraogo et al. 2019 | * | * | * | ** | * | * | * | 9 |
| Worku et al. 2022 | * |  |  | ** | * | * | * | 8 |
| Kebede et al. 2020 | * | * | * | ** | * | * | * | 9 |
| Worku et al. 2022 | * |  |  | ** | * | * | * | 7 |
| Derbew et al. 2024 | * |  |  | ** | * | * | * | 7 |
| Travassos et al. 2016 | * |  | * | ** | * | * | * | 8 |
| Adane et al. 2021 | * |  | * | ** | * | * | * | 8 |
| Solomon et al. 2021 | * | * | * | ** | * | * | * | 9 |
| Arsenault et al. 2021 |  |  |  | ** | * | * | * | 6 |
| Chekol et al. 2023 | * | * | * | ** | * | * | * | 9 |
| Haftu et al. 2021 | * | * | * | * | * | * | * | 8 |
| Getachew et al. 2022 | * | * | * | ** | * | * | * | 9 |
| Ayele et al. 2021 |  |  |  | ** | * | * | * | 5 |
| Gobena et al. 2022 | * | * | * | ** | * | * | * | 9 |
| Tilahun B et al. 2021 |  |  |  | ** | * | * | * | 5 |
| Tilahun B et al. 2022 | * |  | * | ** | * | * | * | 7 |
| Madebo et al. 2021 | * | * | * | ** | * | * | * | 9 |
| Wordofa et al. 2022 | * |  |  | ** | * | * | * | 7 |
| Gebreslassie et al. 2020 |  |  |  |  | * | * | * | 3 |
| Adege et al. 2022 | * |  | * | ** | * | * | * | 8 |
| Kassa 2021 | * | * | * | ** | * | * | * | 9 |
| Nesru et al. | * | * | * | ** | * | * | * | 9 |

Very Good Studies: 9-10 points

Good Studies: 7-8 points

Satisfactory Studies: 5-6 points

Unsatisfactory Studies: 0 to 4 points
